# Stereotactic Body Radiation Therapy for the Treatment of Locally Recurrent and Oligoprogressive Non-Small Cell Lung Cancer: A Single Institution Experience

**DOI:** 10.3389/fonc.2022.870143

**Published:** 2022-05-24

**Authors:** Leah M. Katz, Victor Ng, S. Peter Wu, Sherry Yan, David Grew, Samuel Shin, Nicholas W. Colangelo, Allison McCarthy, Harvey I. Pass, Abraham Chachoua, Peter B. Schiff

**Affiliations:** ^1^ Department of Radiation Oncology, New York University (NYU) Langone Health, Perlmutter Cancer Center, New York, NY, United States; ^2^ Department of Cardiothoracic Surgery, New York University (NYU) Langone Health, Perlmutter Cancer Center, New York, NY, United States; ^3^ Department of Medicine, Division of Medical Oncology, New York University (NYU) Langone Health, Perlmutter Cancer Center, New York, NY, United States

**Keywords:** stereotactic body radiation therapy (SBRT), oligometastasis, NSLC, lung cancer, radiation therapy

## Abstract

**Objectives:**

To investigate the efficacy and safety of lung stereotactic body radiation therapy (SBRT) for non-small cell lung cancer (NSCLC) including oligorecurrent and oligoprogressive disease.

**Methods:**

Single-institution retrospective analysis of 60 NSCLC patients with 62 discrete lesions treated with SBRT between 2008 and 2017. Patients were stratified into three groups, including early stage, locally recurrent, and oligoprogressive disease. Group 1 included early stage local disease with no prior local therapy. Group 2 included locally recurrent disease after local treatment of a primary lesion, and group 3 included regional or well-controlled distant metastatic disease receiving SBRT for a treatment naive lung lesion (oligoprogressive disease). Patient/tumor characteristics and adverse effects were recorded. Local failure free survival (LFFS), progression free survival (PFS), and overall survival (OS) were estimated using the Kaplan Meier method.

**Results:**

At median follow-up of 34 months, 67% of the study population remained alive. The estimated 3-year LFFS for group 1, group 2, and group 3 patients was 95% (95% CI: 86%-100%), 82%(62% - 100%), and 83% (58-100%), respectively. The estimated 3-year PFS was 59% (42-83%), 40% (21%-78%), and 33% (12%-95%), and the estimated 3-year OS was 58% (41-82%), 60% (37-96%), and 58% (31-100%)), respectively for each group. When adjusted for age and size of lesion, no significant difference in OS, LFFS, and PFS emerged between groups (p > 0.05). No patients experienced grade 3 to 5 toxicity. Eighteen patients (29%) experienced grade 1 to 2 toxicity. The most common toxicities reported were cough and fatigue.

**Conclusions:**

Our data demonstrates control rates in group 1 patients comparable to historical controls. Our study also reveals comparable clinical results for SBRT in the treatment of NSCLC by demonstrating similar rates of LFFS and OS in group 2 and group 3 patients with locally recurrent and treatment naïve lung lesion with well-controlled distant metastatic disease.

## Introduction

Lung cancer is the leading cause of cancer death in America, with approximately 225,000 new diagnoses and 160,000 deaths in the United States each year (1). Lung stereotactic body radiotherapy (SBRT) is a non-invasive radiation treatment consisting of highly precise radiation delivered in three to five fractions, and is the standard therapy in inoperable patients with early-stage disease. SBRT achieves local control rates over 90%, overall survival rates comparable to surgery, and low rates of treatment-related morbidity (2–9).

SBRT warrants exploration of its utility outside of the early-stage setting, as it may be advantageous to both surgery and conventionally fractionated radiotherapy (CFRT) for locally recurrent or metastatic disease. Advances in molecularly targeted and immunotherapies have increased life expectancy in patients with locally advanced and metastatic disease. Patients with well-controlled metastatic disease may benefit from durable local control of a primary lesion, especially in the ‘oligoprogressive’ setting, where only one or a few sites of progression are present, but studies on such patients remain limited. Salazar et al. examined the use of SBRT in a small series of metastatic patients, reporting an 86% local control rate and a median survival of 19 months. Seventy five percent of the deaths in this group was due to disease progression outside the planning target volume (PTV), while only one patient died from PTV failure (10). Such a study highlights the potential for primary tumor control in stage IV NSCLC. Furthermore, in older or medically fragile patients with recurrent or advanced disease, SBRT would be advantageous because it avoids the protracted treatment schedule associated with CFRT and the risk of perioperative mortality associated with surgical intervention.

Early evidence also suggests a benefit for SBRT in the setting of oligometastatic NSCLC. In Gomez et al., local consolidative therapy with radiation or surgery demonstrated a prolonged progression free survival and overall survival compared to maintenance therapy alone, resulting in the study being terminated early (11).The UTSW NSCLC Oligomets study was a Phase II trial that was also stopped early due to interim analysis demonstrating a nearly tripling of the progression-free survival when SBRT was added to standard of care (12). Interim results from the Phase III SINDAS trial, looking at the addition of SBRT before tyrosine kinase inhibitor therapy for epidermal growth factor receptor (EGFR) mutated NSCLC, also found a progression-free survival and overall survival benefit (13). 3- and 4-year local failure rates as low as 4.0% and 7.6% has been reported, with the dominant mode of failure being distance (14). Collectively, the results are supportive of a benefit to local consolidative therapy for oligometastatic NSCLC, though more studies are needed to fully evaluate its potential.

This single institution experience is unique in that it provides real world data on the outcomes of SBRT for NSCLC patients with locally recurrent or metastatic disease with oligoprogression involving the lung, with comparison to SBRT outcomes for early stage primary NSCLC tumors. We sought to evaluate with a limited exploratory retrospective analysis of this data the central hypothesis that performance of SBRT in the treatment of NSCLC in terms of local control, progression, and survival may be different in the early stage, recurrent, and oligoprogessive settings.

## Methods

Authorization was obtained from our institutional review board for this retrospective analysis. The clinical data of 60 patients and 62 lesions treated with SBRT for a primary lung lesion between August 2008 and April 2017 at our institution was retrospectively reviewed. All but six patients had tissue diagnosis of NSCLC confirmed by cytologic or pathologic review at our institution. One patient’s pathological diagnosis was confirmed at an outside institution. Patients included in this review were ≥18 years old with Zubrod performance status 0-2. The same criteria applied to the population in RTOG 0236, allowing for more equitable comparisons between our study group and RTOG 0236. AJCC 7^th^ edition was used for staging patients.

Patients were stratified into three groups in an effort to more accurately analyze the role of SBRT in each individual clinical scenario. Group 1 (the early stage group) consisted of 37 patients with early stage NSCLC with lung parenchymal only disease and without prior radiation treatment to the lung. Group 2 (the locally recurrent group) consisted of 15 patients with locally recurrent disease after prior surgery or radiation therapy. Group 3 (the oligoprogressive group) included 8 patients with overall well-controlled regional nodal or distant metastatic disease with fewer than three sites of progression which included a site of progression in the lungs. Patients on systemic therapy for their NSCLC had it held during SBRT and resumed afterwards. Treatment outcomes, including local failure, progression free survival, and overall survival were recorded for each patient. Six patients with non-biopsy-proven metastatic progression were excluded from PFS analysis because of the presence of a concurrent or prior malignancy, precluding determination of the primary tumor.

Patient, tumor, and current and prior treatments were obtained from available medical records. The volume for each patient’s contoured GTV was measured and recorded, and the volumes were compared between stratification groups. Radiation records were obtained for all patients, and pertinent details recorded. Patient, tumor, and treatment characteristics are summarized in **Table 1**.

**Table 1:** Patient Characteristics.

		Overall		Early Stage		Recurrent		Oligoprogressive	
		n	%	n	%	n	%	n	%
**Total**		62		38		16		8	
**Age**	**Median**	79		81		75		73	
	**Max**	99		99		87		90	
	**Min**	41		60		56		41	
**Sex**	**M**	24	39%	11	29%	7	44%	6	75%
	**F**	38	61%	27	71%	9	56%	2	25%
**Stage at Diagnosis**	**IA**	33	53%	28	74%	5	31%	0	0%
	**IB**	17	27%	9	24%	8	50%	0	0%
	**IIA**	1	2%	0	0%	0	0%	1	13%
	**IIB**	0	0%	0	0%	0	0%	0	0%
	**IIIA**	5	8%	1	3%	3	19%	1	13%
	**IIIB**	1	2%	0	0%	0	0%	1	13%
	**IV**	5	8%	0	0%	0	0%	5	63%
**Location**	**Peripheral**	44	71%	32	84%	7	44%	5	63%
	**Central**	18	29%	6	16%	9	56%	3	38%

All patients were monitored weekly during their SBRT treatments for acute treatment-related toxicities, which were recorded by medical staff. All toxicities were scored and classified according to the Common Terminology Criteria for Adverse Events (CTCAE), version 4.03. We also recorded instances of hospital admissions or toxicities that developed once patients finished treatment, and were then seen at regular follow-up visits. **Table 2** outlines all treatment related toxicities occurring during the course of SBRT.

**Table 2:** Radiation Toxicity.

	Overall	Early Stage		Recurrent		Oligoprogressive	
Toxicity	n	n	CTCAE	n	CTCAE	n	CTCAE
**Total**	18	12		5		2	
**Cough**	6	4	1, 1, 2, 2	2	1, 2	0	
**Fatigue**	5	4	1, 1, 1, 2	1	1	0	
**Dysphagia**	2	1	2	0		1	2
**Rib Fracture**	2	2	2, 2	0		0	
**Esophagitis**	2	0		2	2, 2	1	2
**Pneumonitis**	1	1	2	0		0	

All patients underwent 4D-computed tomography (CT)-guided simulation. RT planning was performed using 3D-CRT Varian Eclipse Versions (version from 2008 to 2011). Treatment volumes were constructed according to the same guidelines used to construct the SBRT fields in RTOG 0236, regardless of the patient’s group. The prescription dose ranged from 40 to 60 Gy, delivered over 3 to 5 fractions, using 6 MV photons. Dose-volume histograms were constructed for doses to the target volume, lungs, hearts, bronchial tree, and ribs, if receiving dose from a proximally targeted lesion. Institutional dose constraints for all of the treated patients represented here are in accordance with RTOG 0236 for the three and five fraction regimens, with the introduction of a rib constraint on September 1, 2012. The rib constraint ensures that for 3 and 5 fractions, 1 cc of rib does not exceed 35.0 Gy and 28.90 Gy, respectively. It is important to note that while our institution formally acknowledges the single rib dose constraint, it is at times compromised when clinically reasonable, in favor of PTV coverage.

Statistical analysis was performed with R version 3.4.2 and was used to generate OS, local failure free survival (LFFS), and progression free survival (PFS) analysis. Three year estimates of survival and local failure were calculated *via* the method of Kaplan Meier. Acute lung toxicities were documented according to the Common Terminology Criteria for Adverse Events, version 4.03. Treatment response was scored according to the RTOG 0236 protocol. Target lesion delineation was based on planning 4D-CT and most recent diagnostic CT or PET-CT.

## Results

Sixty patients and sixty-two tumors were evaluated in this retrospective study. Patient characteristics, as well as staging (AJCC 7th edition), groupings, tumor location, and can be found in **Table 1**. Of the 62 patients, 39% were male, 61% female, and the median age at diagnosis was 79 years (range, 41-99 years). 54 patients (87.1%) had a smoking history, with a mean pack-years of 47.1. Median dose was 50 Gy (range 40-60 Gy) delivered over a median of 5 fractions (range 3-5).

Regarding tumor characteristics, 18 (29%) lesions were located centrally, defined as within 2 cm of the bronchial tree. 46 lesions (74.2%) were histologically classified as adenocarcinoma, eight (12.9%) were classified as squamous cell carcinoma, one (1.6%) was classified as bronchoalveolar carcinoma, and 7 (11.3%) were indeterminate. The volume for each patient’s contoured GTV was measured and recorded, with a conglomerate cohort mean of 11.0 cm (3). For groups 1, 2, and 3, the GTV means were 8.5 cm (3,) 19.7 cm (3,) and 4.9 cm (3,) respectively, which was statistically different (p = 0.03) between the three groups volumes detected on one way ANOVA test.

**Figure 1:**
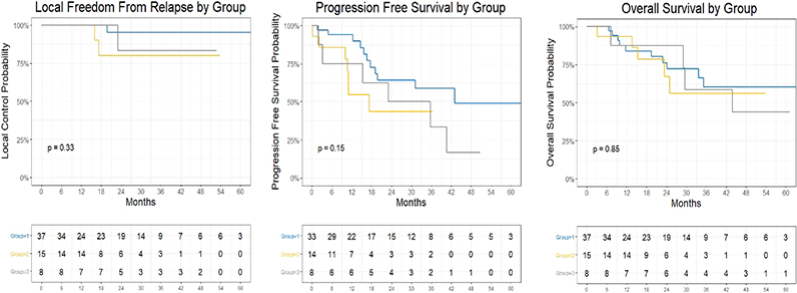
Kaplan Meier Plot of Local Freedom From Relapse by Group, Progression Free Survival by Group, and Overall Survival by Group.

Median follow-up, taken from the date of the initial SBRT fraction to the date of last follow-up among survivors, was 34 months, at which time the 67% of all patients remained alive. For group 1, 2, and 3 patients, the Kalpan-Meier estimated 3-year LFFS was 95% (95% CI: 86%-100%), 82%(62% - 100%), and 83% (58-100%), respectively (**Figure 1**). The estimated 3-year PFS was 59% (42-83%), 40% (21%-78%), and 33% (12%-95%), and the estimated 3-year OS was 58% (41-82%), 60% (37-96%), and 58% (31-100%)), respectively for each group.

Treatment was relatively well tolerated by the cohort population. 18 patients (29%) developed grade 1-2 toxicity, including: cough (6), fatigue (5), esophagitis (2), dysphagia (2), rib fracture (2), and pneumonitis (1). No patients developed grade 3-4 toxicity. See **Table 2**. There have been no rib fractures since adoption of the rib constraint at our institution in September 2012. There were no recorded cases of treatment related deaths, inter-fractional treatment delays, or early treatment stops secondary to toxicity.

## Discussion

SBRT has emerged as a valuable treatment modality for managing lung carcinoma in various clinical scenarios. In this study, we conducted a single-institution retrospective analysis to investigate the efficacy and safety of lung SBRT in a diverse cohort of NSCLC patients. We confirm institutional competency and explore the role of SBRT in locally recurrent and oligoprogressive disease. In early-stage NSCLC patients, we demonstrate local control and overall survival outcomes comparable to historical outcomes. Numerous prospective trials have reported 3-year local control and overall survival rates ranging from 53-92% and 43-60%, respectively, for T1-T2 inoperable patients treated with SBRT (3–7).

Several studies have demonstrated that SBRT achieves superior overall survival *via* improved local control. The role of SBRT for early-stage NSCLC patients is well-established. Patients treated with CFRT for early stage NSCLC most often die from a painful uncontrolled primary lesion, and have dismal overall survival rates ranging from 20-40% at three years (15–17). Studies of dose escalation with conventional fractionation, including the Michigan series and RTOG 9311, have similarly shown unsatisfactory results, reporting a 50% treatment failure and 50-78% locoregional control rate, respectively (18, 19). The CHISEL trial, conducted by the Trans-Tasman Radiation Oncology Group and the Australasian Lung Cancer Trials Group, showed in a randomized trial in T1-T2a NSCLC, superior freedom from local failure (HR = 0.29, p = 0.002) and longer overall survival (HR = 0.51, p = 0.020) with SBRT as compared to CFRT (20). SBRT affords higher rates of local tumor control and overall survival by achieving higher biologically effective doses (BED). Martel et al. estimated through analysis of the University of Michigan phase I dose escalation study that 84.5 Gy was required to achieve 50% 30-month tumor control probability (21), and Onishi et al. found in a retrospective multi-institutional study superior local control and overall survival with BED > 100 Gy in stage I NSCLC treated with SBRT (22). Mehta et al. reviewed 42 studies on CFRT and SBRT in stage I NSCLC, and calculated with linear quadratic (LQ) and universal survival curve (USC) models that a BED of at least either 159 Gy (LQ) or 124 Gy (USC) would be required for 90% tumor control probability (23). Our results build on these findings, and support a role for SBRT for patients with locally recurrent or oligoprogressive disease in the lung. Further prospective investigation is warranted.

Indirect comparisons suggest that SBRT may have comparable performance to surgery for early NSCLC. Although SBRT for early-stage NSCLC has not been directly compared to surgery in a completed randomized trial, RTOG 0403 and RTOG 0618 provided evidence that in operable patients, SBRT results in local control and overall survival rates comparable to that of historical controls receiving surgery (7, 8). The only prospective randomized evidence directly comparing SBRT to surgery is a pooled analysis of the STARS and ROSEL studies, which suggested a survival advantage for operable patients receiving SBRT over those who received surgical resection (HR 0.14, p = 0.037), and reported more treatment-related adverse effects in the surgery group (9). The survival curve separation favoring radiotherapy in this early pooled analysis is a well-recognized phenomenon, and has been attributed in both population-based and prospective randomized data to perioperative mortality associated with resection (24–27). However, this advantage may be counteracted by the lack of regional nodal therapy provided by SBRT. Whether the superior treatment-related mortality of SBRT or the potential for superior oncologic control with nodal resection is more important in determining overall survival in NSCLC will need to be assessed in prospective trials comparing both modalities. Patients with locally recurrent or well-controlled metastatic disease and a growing lung lesion will likely benefit less from regional nodal therapy, and hence represent clinical niches well-suited for SBRT. The data presented here support these findings, and also suggest that SBRT may also achieve comparable local control to surgery for more advanced stages of disease.

Our results for SBRT in oligoprogressive NSCLC are consistent with the general literature for oligometastatic disease. The Phase II UTSW trial, with an average 9.6 month follow-up due to early closure due to interim analysis, had median 9.7 month progression-free survival and no local failures with the addition SABR, compared to 3.5 months without (15). Our retrospective study had an 3 year progression free survival and overall survival for oligoprogressive NSCLC estimated around 35% and 58%, respectively are similar to results of Gomez et al. (14) Our study also had no local failures in the first year after treatment. Further work, including Phase III trials results, will be needed to better evaluate the benefit of SBRT in this setting. This includes subgroup analysis to evaluate for differences in outcomes for different histologies and the influence of smoking history. The Phase II/III NRG-LU002 trial, examining the benefit of adding SBRT for oligometastatic NSCLC, completed accrual for its Phase II portion in late 2021, and may open after interim analysis for the planned Phase III portion to evaluate the benefit of adding SBRT to standard maintenance therapy (28). The SARON trial is a phase III trial also evaluating the effectiveness of SBRT in addition to standard chemotherapy (29).

The limitations of this study include its retrospective nature, which could introduce biases in the data. The small number of patients further limits the conclusions that can be drawn. Nevertheless, our data is sufficient to suggest novel clinical niches for the application of SBRT. The broadening of lung SBRT’s use is especially important given the success of targeted systemic and immunotherapies, which has created an expanding cohort of well-controlled recurrent and advanced stage lung cancer patients who may benefit from durable local control. Additionally, earlier detection of lung cancer is leading to increasing numbers of locally-treated early-stage disease for which SBRT is an excellent and well-tolerated salvage option.

As we continue to perfect the delivery of SBRT, our ability to obtain local control and concomitant mitigation of treatment related morbidity will continue to improve. Lung SBRT is an important targeted treatment option in the management of an increasing number of clinical lung cancer presentations. It is upon the Radiation Oncology community to innovate new applications for and to optimize the delivery of SBRT.

## Data Availability Statement

The raw data supporting the conclusions of this article will be made available by the authors, without undue reservation.

## Ethics Statement

The studies involving human participants were reviewed and approved by NYU Grossman School of Medicine IRB. Written informed consent for participation was not required for this study in accordance with the national legislation and the institutional requirements.

## Author Contributions

PS- experimental design, treatment, data collection, data analysis, manuscript preparation. LK- data collection, data analysis, and manuscript preparation. VN- data collection, data analysis, manuscript preparation. PW- data analysis, manuscript preparation. SY- data collection, data analysis, manuscript preparation. DG- data collection, data analysis, manuscript preparation. SS- data collection, data analysis, manuscript preparation. NC- data analysis, manuscript preparation. AM- data analysis, manuscript preparation. HP- data analysis, manuscript preparation. AC- data analysis, manuscript preparation. All authors contributed to the article and approved the submitted version.

## Funding

Biostats Shared Resource of the Laura and Isaac Perlmutter Cancer Center (P30 CA016087), NYU Langone Health.

## Conflict of Interest

The authors declare that the research was conducted in the absence of any commercial or financial relationships that could be construed as a potential conflict of interest.

## Publisher’s Note

All claims expressed in this article are solely those of the authors and do not necessarily represent those of their affiliated organizations, or those of the publisher, the editors and the reviewers. Any product that may be evaluated in this article, or claim that may be made by its manufacturer, is not guaranteed or endorsed by the publisher.
